# Ultrasensitive on-site detection of aflatoxin M_1_ in milk using a chitosan-MWCNT-graphene nanocomposite aptasensor with sub-regulatory limit capability

**DOI:** 10.1038/s41598-026-38492-w

**Published:** 2026-02-05

**Authors:** Romina Vahab Zadeh, Ali Mohamadi Sani, Vahid Hakimzadeh, Afshin Farahbakhsh

**Affiliations:** 1 Department of Food Science and Technology, Qu.C., Islamic Azad University, Quchan, Iran; 2 Department of chemical engineering, Qu.C., Islamic Azad University, Quchan, Iran

**Keywords:** Electrochemical aptasensor, AFM_1_, MWCNT/graphene, Chitosan film, Dairy safety monitoring, Biochemistry, Biotechnology, Chemistry, Materials science, Nanoscience and technology

## Abstract

This study presents a novel electrochemical aptasensor for ultrasensitive detection of aflatoxin M_1_ (AFM_1_) in milk samples, utilizing a gold electrode modified with chitosan-functionalized multiwalled carbon nanotube/graphene nanocomposite (CS/f-MWCNTs-Gr). The platform was fabricated through covalent immobilization of an amino-modified aptamer onto the nanocomposite surface, enhancing electronic transmission and biorecognition efficiency. Cyclic voltammetry (CV) confirmed stepwise electrode modification, while square wave voltammetry (SWV) quantified AFM_1_ via suppression of the [Fe(CN)_6_]^3−/4−^ redox signal upon target binding. Under optimized conditions, the sensor demonstrated a wide linear range (1–1000 nM) covering EU/US regulatory limits, an ultra-low detection limit (0.03 nM, 9.8 ng/L, below EU regulatory limit of 25 ng/kg), and exceptional specificity (> 90% signal suppression against interferents at 10-fold higher concentrations). The aptasensor exhibited high reproducibility (RSD = 5.4%, *n* = 5) and retained 92% signal after 14-day storage. Validated in commercial milk, it achieved 96–106% recoveries with RSD < 4.9% (*n* = 5), outperforming reference methods in precision and practicality. This cost-effective platform shows significant potential for on-site monitoring of mycotoxins in dairy products.

## Introduction

 Aflatoxins (AFs) are a group of toxic secondary metabolites primarily produced by *Aspergillus flavus* and *Aspergillus parasiticus*^1^. Among them, aflatoxin B_1_ (AFB_1_) is the most potent and carcinogenic compound, which can be metabolized to aflatoxin M_1_ (AFM_1_) in the liver of dairy animals following ingestion of contaminated feed^2–4^. AFM_1_ is subsequently excreted into milk and is known for its chemical stability during pasteurization and other thermal processing steps^5,6^. Even at trace levels, AFM_1_ poses serious health risks, including mutagenic, carcinogenic, teratogenic, genotoxic, and immunosuppressive effects^7^. Owing to these concerns, AFM_1_ has been classified as a Group 1 carcinogen by the International Agency for Research on Cancer (IARC)^8,9^. Regulatory authorities such as the European Union and the United States Food and Drug Administration have set maximum residue limits of 25 ng/kg and 0.5 µg/L for AFM_1_ in milk, respectively^10^.

Conventional analytical techniques for AFM_1_ detection, including high-performance liquid chromatography (HPLC)^11,12^, liquid chromatography–mass spectrometry (LC–MS)^13^, enzyme-linked immunosorbent assay (ELISA)^14,15^, colorimetry^16^, and fluorescence-based methods^17,18^, offer varying degrees of sensitivity and specificity. However, they are often constrained by limitations such as time consumption, laborious procedures, and high operational costs. Electrochemical sensors have emerged as promising alternatives due to their simplicity, rapid response, high sensitivity, and low cost^19,20^.

Aptamers are the synthetic single-stranded DNA or RNA molecules selected through SELEX (Systematic Evolution of Ligands by Exponential enrichment), offer high binding affinity, stability, and ease of synthesis compared to antibodies^21–24^. As a result, they have become valuable recognition elements in a wide range of biosensing platforms, including fluorescence^25^, colorimetric^26^, surface-enhanced Raman spectroscopy^27^, surface plasmon resonance^28^, and electrochemical aptasensors^29^. Among these, electrochemical aptasensors are particularly attractive for their signal amplification capability, cost-efficiency, and compatibility with portable devices^30–32^.

To further enhance sensor performance, nanomaterials such as multiwalled carbon nanotubes (MWCNTs) and graphene have been widely used due to their high surface area, excellent electrical conductivity, and ability to facilitate biomolecule immobilization^33–38^. Chitosan (CS), a biocompatible polymer, serves as an effective matrix for aptamer immobilization. Although CS is inherently non-conductive, its integration with conductive nanomaterials like MWCNTs and graphene yields a hybrid substrate suitable for electrochemical biosensing applications^39,40^.

Despite the extensive efforts to develop electrochemical aptasensors for AFM_1_, most reported platforms rely on single-component nanostructures or simple physical adsorption of aptamers on electrode surfaces, which often suffer from limited electron‑transfer efficiency, poor stability, and insufficient aptamer loading capacity. In addition, several previously reported AFM_1_ electrochemical aptasensors provide relatively narrow linear ranges and have only been partially validated in real milk samples. In contrast, only a few studies have explored the synergistic integration of chitosan with multiwalled carbon nanotubes and graphene on a gold electrode using a covalent immobilization strategy. Such a multifunctional hybrid interface can simultaneously enhance conductivity, provide a biocompatible matrix, and enable stable, high‑density aptamer attachment. Therefore, the development of a CS/MWCNT‑graphene composite–modified gold electrode in this work represents a novel sensing architecture that overcomes major limitations of existing AFM_1_ aptasensors and enables improved analytical performance, including a wide linear range and sensitive, reliable determination of AFM_1_ in commercial milk samples.

In this study, we report the fabrication of a novel electrochemical aptasensor for ultra-sensitive and selective detection of AFM1 in milk. The sensor was constructed via covalent immobilization of an amino-terminal aptamer onto a CS/MWCNT-graphene composite film modified gold electrode. The resulting platform is expected to offer enhanced analytical performance through improved electron transfer and increased aptamer binding capacity.

## Materials and methods

### Chemicals

A 21-mer amino-modified DNA aptamer (5′-NH_2_-ACTGCTAGAGATTTTCCACAT-3′), bearing a free amine group at the 5′ end, was obtained from Topazgene (Iran). Prior to use, a stock solution of the aptamer was prepared in Tris–HCl buffer (pH 7.4) and stored at − 20 °C until further experiments (see^8^ for similar preparation). Multiwalled carbon nanotubes (MWCNTs) and graphene nanosheets were supplied by US Research Nanomaterials (USA). Chitosan, glutaraldehyde, aflatoxins M_1_ and B_1_, and ochratoxin B were purchased from Sigma-Aldrich (USA). All other reagents and solvents were of analytical grade and were obtained from Merck Chemical Company (Germany) and used without further purification. Deionized water was used throughout for all solution preparations.

### Apparatus

Electrochemical measurements were conducted using a conventional three-electrode system comprising a gold working electrode, an Ag/AgCl (saturated KCl) reference electrode, and a platinum wire counter electrode. The system was connected to a µ-Autolab Type III potentiostat/galvanostat and operated using NOVA software (Metrohm, The Netherlands). The pH of all solutions was adjusted and monitored using a Metrohm pH meter (Model 827). Surface morphologies of the modified and unmodified electrodes were characterized by scanning electron microscopy (SEM) using a LEO 1450 VP instrument (Carl Zeiss, Germany) (as previously described in^35^.

### Fabrication of aptamer/CS/f-MWCNTs-Gr/AuE

**Fig. 1:**
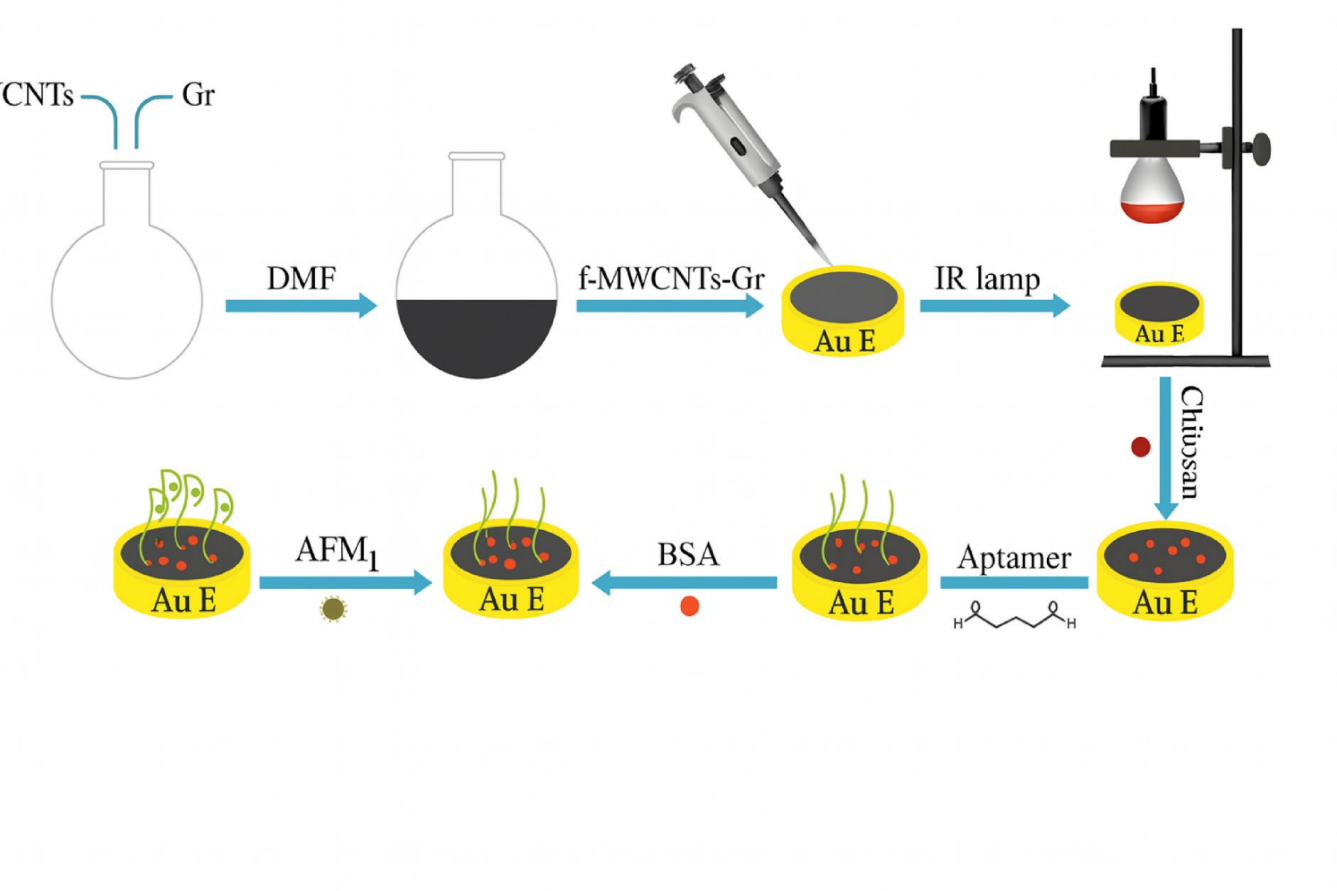
Fabrication process of the electrochemical aptasensor. Schematic illustration showing stepwise modification of the gold electrode with (1) f-MWCNTs-Gr nanocomposite, (2) chitosan film, (3) aptamer immobilization via glutaraldehyde crosslinking, and (4) BSA blocking.

The fabrication process of the electrochemical aptasensor is schematically illustrated in Fig. 1. Initially, multi-walled carbon nanotubes (MWCNTs) were functionalized with carboxylic acid (–COOH) groups. For this purpose, 0.3 g of MWCNTs was refluxed in 50 mL of concentrated nitric acid at 120 °C for 24 h. The resulting suspension was filtered and washed repeatedly with deionized water until the pH reached approximately 7.0, followed by vacuum drying at 60 °C for 12 h.

Prior to electrode modification, the gold electrode (AuE) was mechanically polished using an alumina slurry, then sonicated in both water and ethanol baths to remove surface contaminants. Electrochemical cleaning was subsequently performed by potential cycling in 1.0 M H_2_SO_4_ solution between – 0.2 V and + 1.5 V.

For surface functionalization, 5 µL of a nanocomposite suspension-prepared by dispersing 5 mg of functionalized MWCNTs and 5 mg of graphene in 5 mL of dimethylformamide (DMF)-was drop-cast onto the pretreated AuE surface. The solvent was evaporated by infrared (IR) lamp irradiation. After drying, 5 µL of 1 mg/mL chitosan solution was cast onto the modified surface and allowed to dry.

To generate biorecognition sites, the CS/f-MWCNTs-Gr-modified electrode was first incubated in a 2% glutaraldehyde solution to introduce reactive aldehyde groups on the chitosan layer, enabling covalent coupling with the amino‑modified aptamer, followed by incubation in Tris–HCl buffer (pH 8.0) containing 1 µM of amino-modified aptamer for 1 h at room temperature. To block unreacted binding sites and minimize nonspecific adsorption, 5 µL of 0.2% bovine serum albumin (BSA) solution was added to the modified surface. The final platform is referred to as aptamer/CS/f-MWCNTs-Gr/AuE^24^.

### Electrochemical measurements

All electrochemical measurements were performed in a probe solution containing 0.1 M KCl and 5 mM [Fe(CN)_6_]^3−/4−^ as a redox couple. The peak currents of the [Fe(CN)_6_]^3−/4−^ system were recorded in the absence and presence of AFM_1_, and the variation in current response was employed to quantify AFM_1_ in the solution.

For the binding assay, 5 µL of AFM_1_ solution was dropped onto the surface of the aptamer/CS/f-MWCNTs-Gr-modified electrode and incubated for 40 min at room temperature to allow target-aptamer interaction.

In the absence of AFM_1_, the aptamer remains in an unfolded, flexible conformation, allowing unhindered electron transfer between the redox probe and the electrode surface. Upon target binding, the aptamer undergoes a conformational change-likely forming a folded or G-quadruplex structure that partially blocks electron transfer pathways, resulting in a significant decrease in the peak current of the [Fe(CN)_6_]^3−/4−^ couple^30^.

### Sample preparation

Raw milk samples were purchased from a local supermarket in Mashhad, Iran. For sample preparation, 5.0 mL of milk was mixed with 20 mL of Tris-HCl buffer solution (pH 8.0) and sonicated for 30 min to ensure proper dispersion and protein denaturation. The mixture was subsequently shaken for 10 min, then centrifuged at 10,000 rpm for 15 min to remove fat globules. The supernatant was collected and diluted further with Tris-HCl buffer. The resulting solution was directly used for the electrochemical determination of AFM_1_^41^.

### FTIR analysis

Fourier transform infrared (FTIR) spectra of the CS‑f‑MWCNTs‑Gr nanocomposite were recorded in the wavenumber range 4000 –400 cm^−1^ using an FTIR spectrometer (Nicolet iS10, Thermo Scientific, USA) equipped with an ATR accessory. The spectra were used to confirm the presence of oxygen‑ and nitrogen‑containing functional groups in the modified nanocomposite film.

### Ethics declaration

This study exclusively utilized commercially purchased milk samples from retail markets. No human participants or live animals were involved in the research. Therefore, ethical approval was not required under institutional guidelines. All experiments complied with standard analytical safety protocols.

## Results and discussion

### Morphological characterization of the modified electrode

**Fig. 2:**
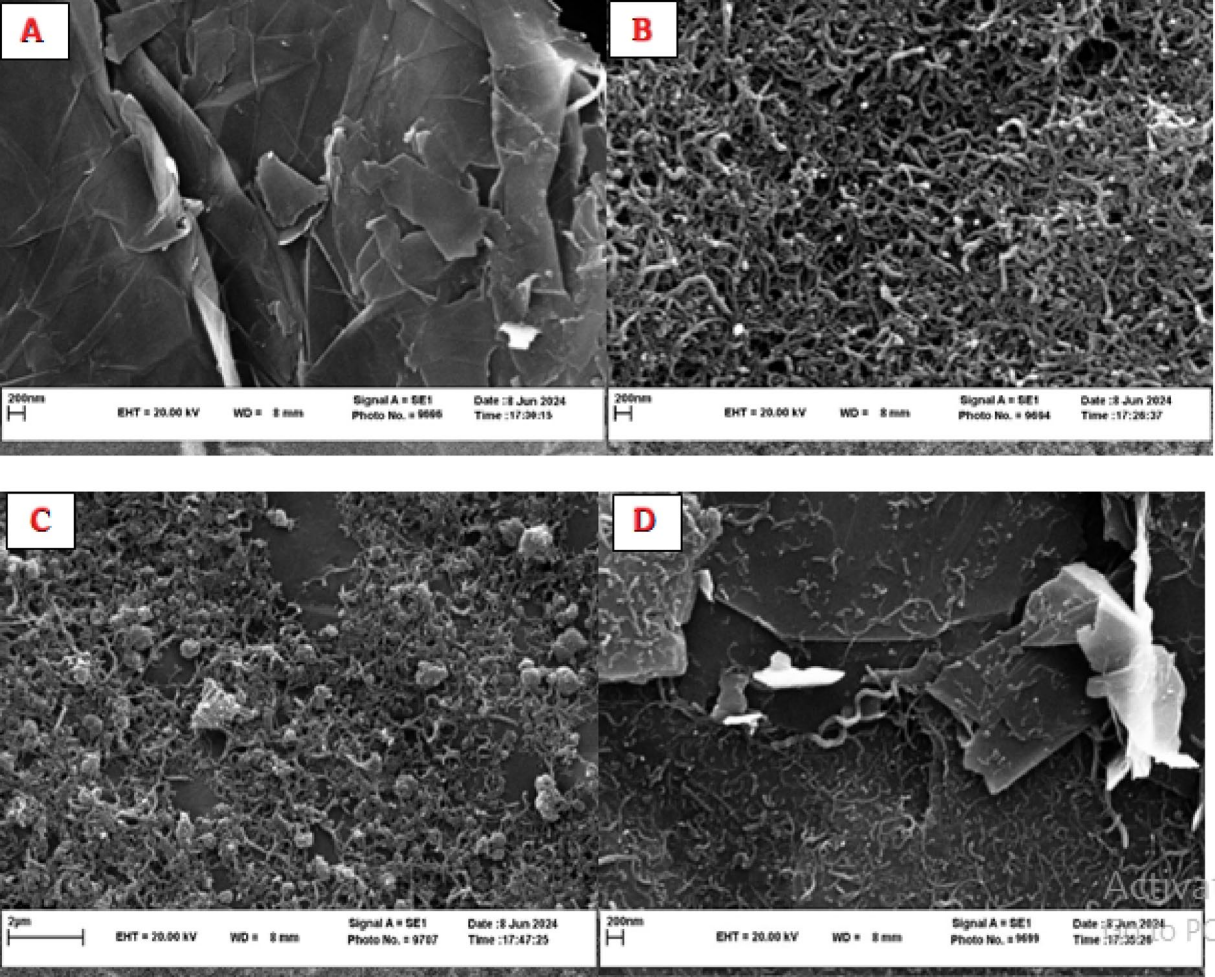
SEM characterization of electrode modifications. (**A**) Functionalized MWCNTs on AuE showing tubular morphology. (**B**) Graphene nanosheets on AuE exhibiting wrinkled sheet structure. (**C**) f-MWCNTs-Gr nanocomposite demonstrating homogeneous integration. (**D**) Chitosan-encapsulated CS/f-MWCNTs-Gr nanocomposite forming a continuous biointerface.

The morphological evolution of the electrode surface during modification was analyzed by SEM (Fig. 2). Figure 2A reveals the characteristic tubular morphology of functionalized MWCNTs, while Fig. 2B shows graphene’s distinctive wrinkled nanosheet structure. The f-MWCNTs/Gr nanocomposite (Fig. 2C) demonstrates homogeneous integration with preserved porosity, creating an interconnected conductive network. Crucially, chitosan modification (Fig. 2D) yields a continuous uniform film that encapsulates the nanocomposite while maintaining structural integrity. This morphological transition from porous nanostructures (Figs. 2A–C) to a consolidated biointerface (Fig. 2D) enhances biocompatibility for aptamer immobilization and improves electrochemical stability. This observation is consistent with previous reports on chitosan-nanocarbon composites^35,39^.

### FTIR characterization

**Fig. 3:**
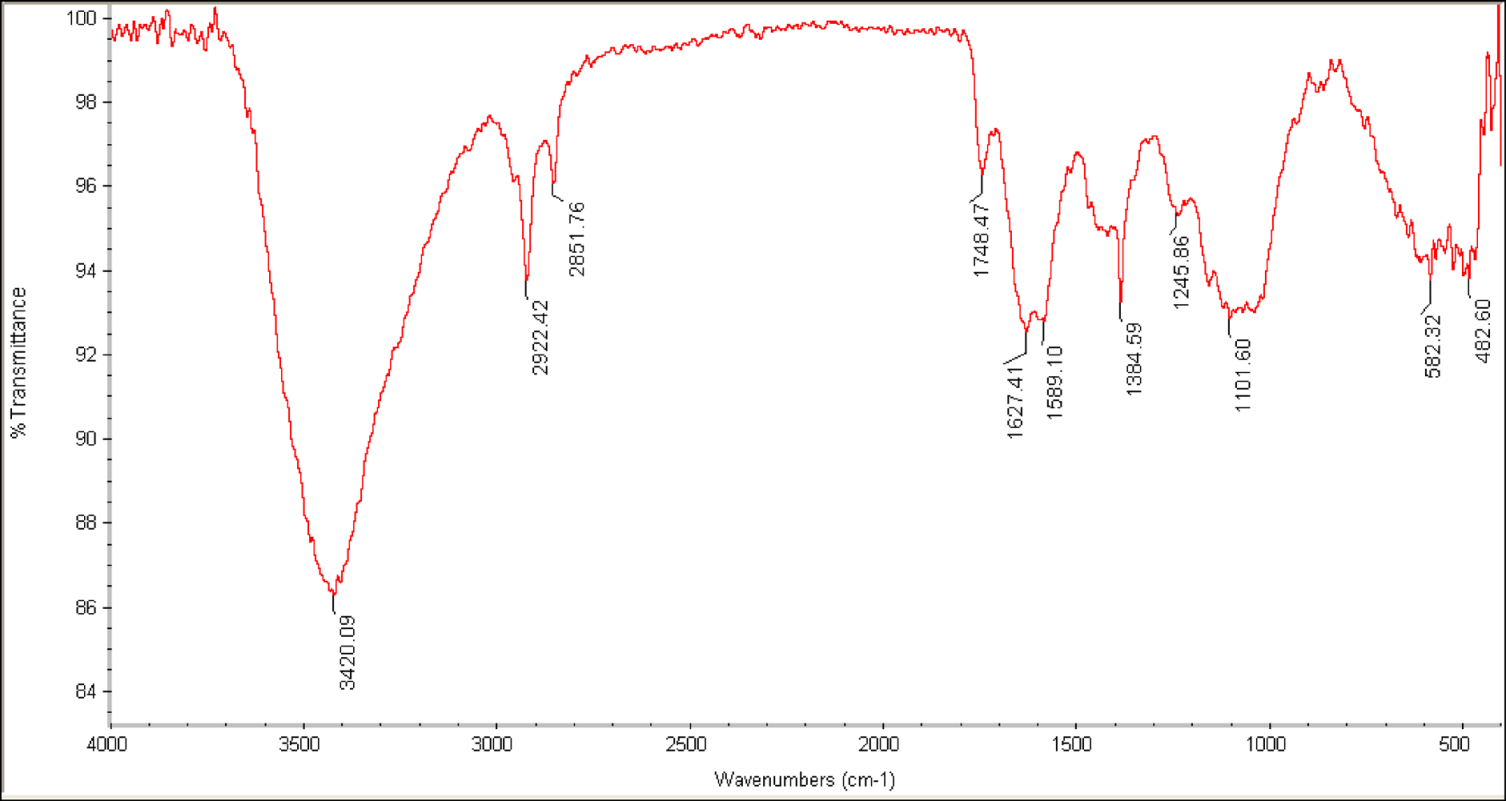
FTIR spectrum of the CS‑f‑MWCNTs‑Gr nanocomposite film. Confirming the presence of oxygen‑ and nitrogen‑containing functional groups on the modified sensing layer.

As shown in Fig. 3, the FTIR spectrum of the CS-f-MWCNTs-Gr nanocomposite exhibits a broad O–H/N–H band around 3420 cm^−1^, C–H stretching bands at 2922 and 2851 cm^−1^, and a clear C=O band near 1748 cm^−1^, together with amide/aromatic and C–O–C/C–O vibrations at lower wavenumbers. These features are consistent with the presence of oxidized MWCNTs, chitosan and glutaraldehyde-derived linkages, and thus corroborate the proposed covalent immobilization of the amino-terminated aptamer on the modified electrode surface.

### Electrochemical characterization of the aptasensor

**Fig. 4:**
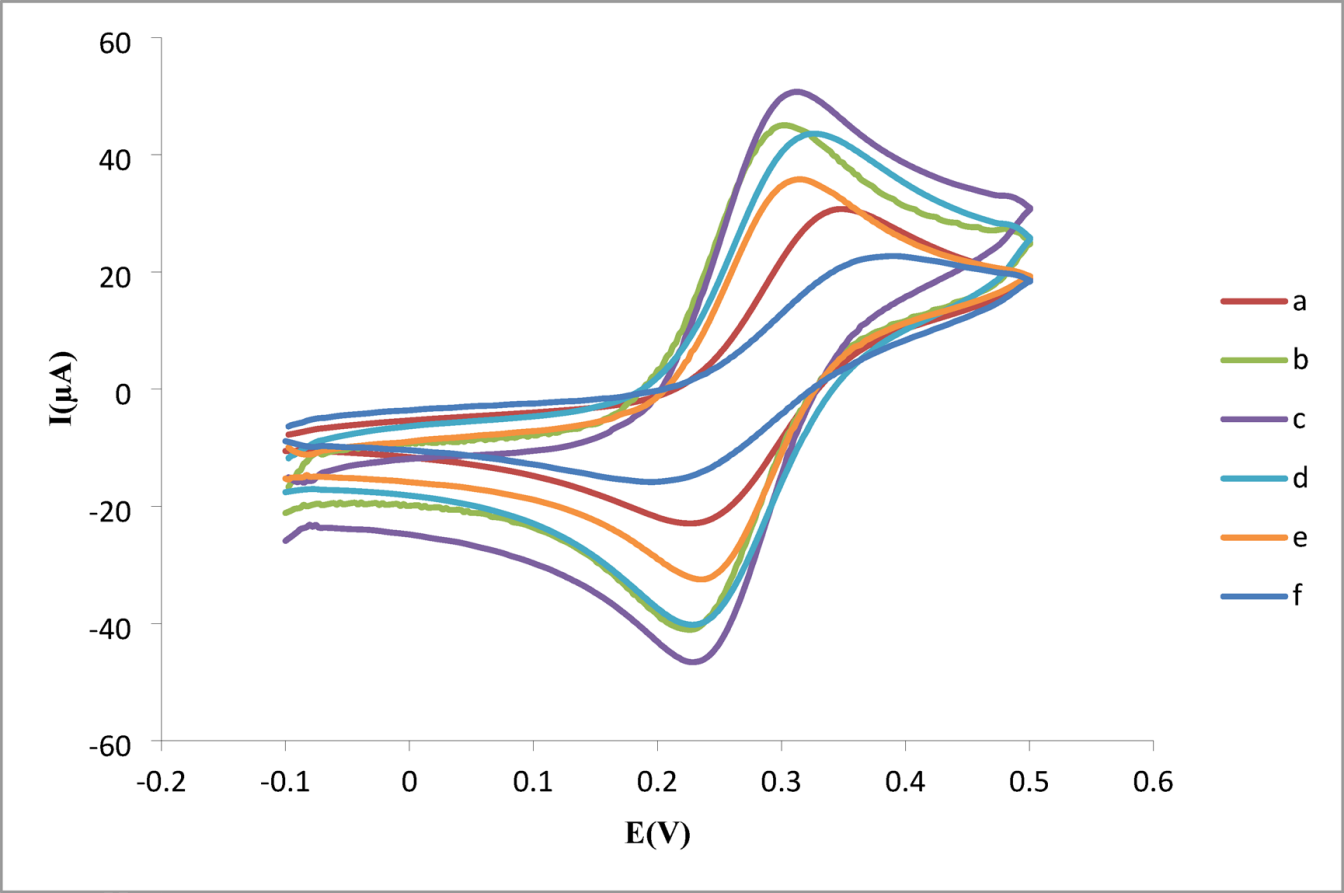
Electrochemical characterization of modification steps. Cyclic voltammograms in 0.1 M KCl/5 mM [Fe(CN)_6_]^3−/4−^: (a) Bare AuE, (b) f-MWCNTs/AuE, (c) f-MWCNTs-Gr/AuE, (d) CS/f-MWCNTs-Gr/AuE, (e) aptamer/CS/f-MWCNTs-Gr/AuE, (f) After AFM_1_ binding. Conditions: − 0.1 to + 0.5 V scan range, 50 mV/s scan rate.

Cyclic voltammetry (CV) was employed to monitor each step of electrode modification using 5 mM [Fe(CN)_6_]^3−/4−^ in 0.1 M KCl as a redox probe (Fig. 4). The bare gold electrode (curve a) exhibited low redox peak currents, indicating sluggish electron transfer kinetics. Modification with f-MWCNTs (curve b) increased peak currents by 210% due to enhanced conductivity and surface area, consistent with carbon nanomaterial behavior^35^. Further improvement with f-MWCNTs/Gr nanocomposite (curve c) amplified currents by 320%, outperforming graphene-only electrodes by 40%^37^, confirming synergistic electron transport between 1D nanotubes and 2D nanosheets.

Critically, chitosan deposition (curve d) reduced currents by 37% (vs. curve c), confirming its insulating properties, yet proving successful nanocomposite encapsulation essential for stable aptamer anchoring^39^. Subsequent aptamer immobilization (curve e) caused a 52% current drop exceeding the 30–40% suppression in AuNP-based systems^8^ demonstrating superior biorecognition layer density. Finally, AFM_1_ binding (curve f) triggered an additional 28% decrease, attributed to G-quadruplex formation blocking electron transfer^7^. This stepwise signal attenuation validates both successful biosensor assembly and target-responsive operation.

### Optimization of analytical parameters for aptasensor performance

To enhance the analytical performance of the developed electrochemical aptasensor, key experimental variables, including the ratio of f-MWCNTs to graphene, the loading volumes of nanocomposite and chitosan, aptamer concentration, and target incubation time, were systematically optimized.

### Effect of f-MWCNTs to Gr ratio

**Fig. 5:**
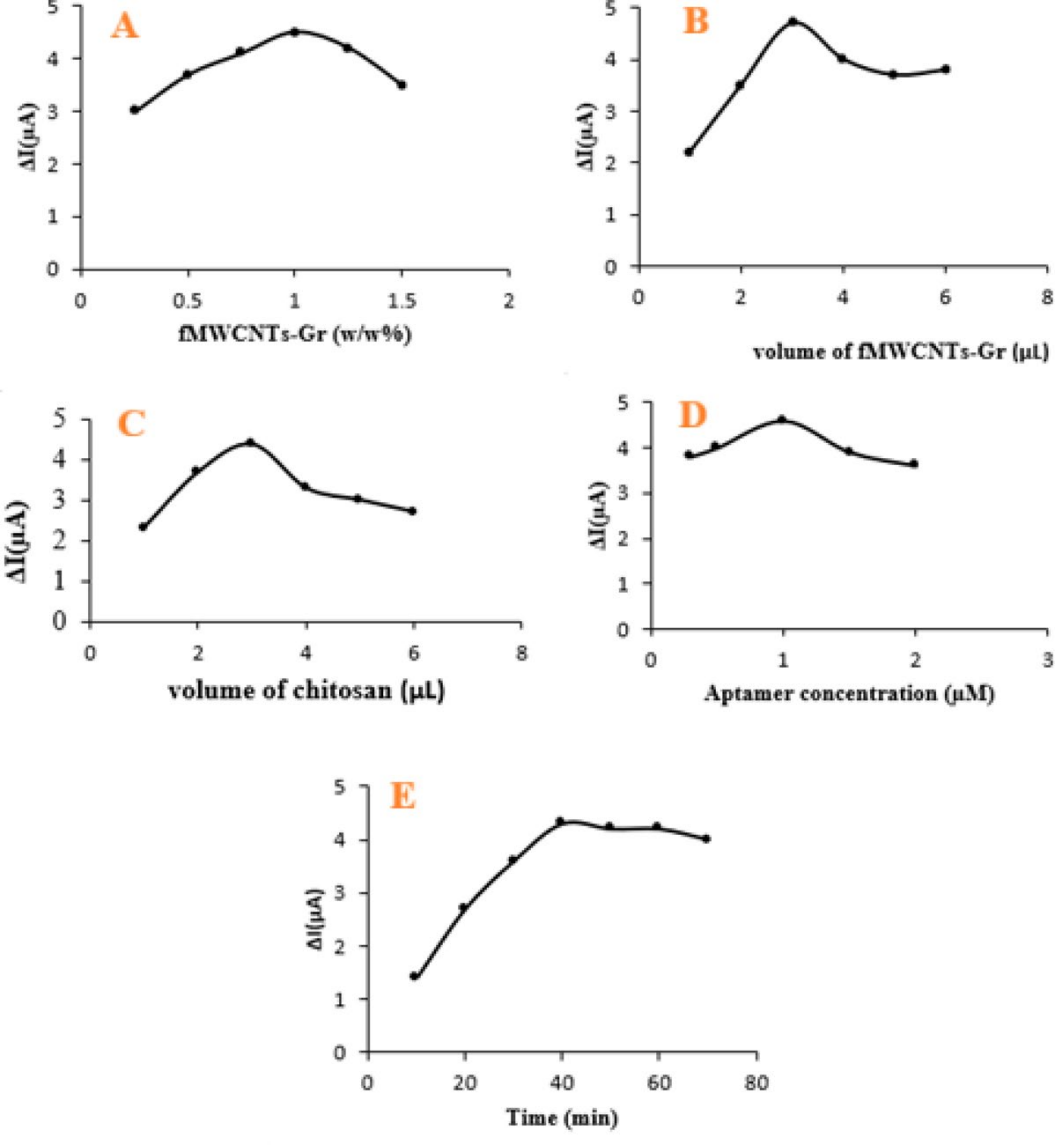
Optimization of aptasensor parameters. (**A**) f-MWCNTs: Gr mass ratio (1:1 optimal). (**B**) f-MWCNTs-Gr nanocomposite volume (3 µL optimal). (**C**) Chitosan volume (3 µL optimal). (**D**) Aptamer concentration (1.0 µM optimal). (**E**) AFM_1_ incubation time (40 min optimal).

The ratio of f-MWCNTs to graphene is critical in determining the conductivity and effective surface area of the sensing interface. Various ratios (w/w) were evaluated, and the maximum peak current was obtained at a 1:1 ratio, as shown in Fig. 5A. This optimal ratio likely offers the best synergistic effect between the 1D tubular and 2D sheet nanostructures, facilitating electron transfer and aptamer immobilization.

### Effect of f-MWCNTs-Gr and chitosan volume

The volumes of both the f-MWCNTs-Gr nanocomposite and chitosan were varied (1–5 µL) to evaluate their influence on sensor response. As illustrated in Fig. 5B and C, peak currents increased with increasing volume up to 3 µL but declined at higher volumes. This behavior may result from excessive film thickness, which impedes electron transfer due to higher internal resistance. Therefore, 3 µL was selected as the optimal volume for both components.

### Effect of aptamer concentration

The surface coverage and target recognition efficiency are directly influenced by aptamer concentration. The response of the aptasensor was examined across a concentration range of 0.5–2.0 µM. As shown in Fig. 5D, peak current increased to 1.0 µM, suggesting enhanced aptamer density and binding efficiency. Beyond this point, current decreased, possibly due to steric hindrance or multilayer formation. Thus, 1.0 µM was selected as the optimum aptamer concentration.

### Effect of incubation time with AFM_1_

The interaction time between AFM_1_ and the immobilized aptamer was investigated in the range of 10–60 min. As shown in Fig. 5E, the peak current decreased steadily up to approximately 40 min and then reached a plateau, indicating saturation of the available binding sites and that the aptamer AFM_1_ interaction at the electrode surface was close to equilibrium. Therefore, an incubation time of 40 min was selected as a compromise between achieving equilibrium binding and maintaining an acceptable total assay duration. It is worth noting that similar incubation periods in the range of 30–60 min have also been reported for electrochemical (apta)sensors for mycotoxin detection, confirming that such assay times are realistic and practical^42,43^.

### Calibration curve

**Fig. 6:**
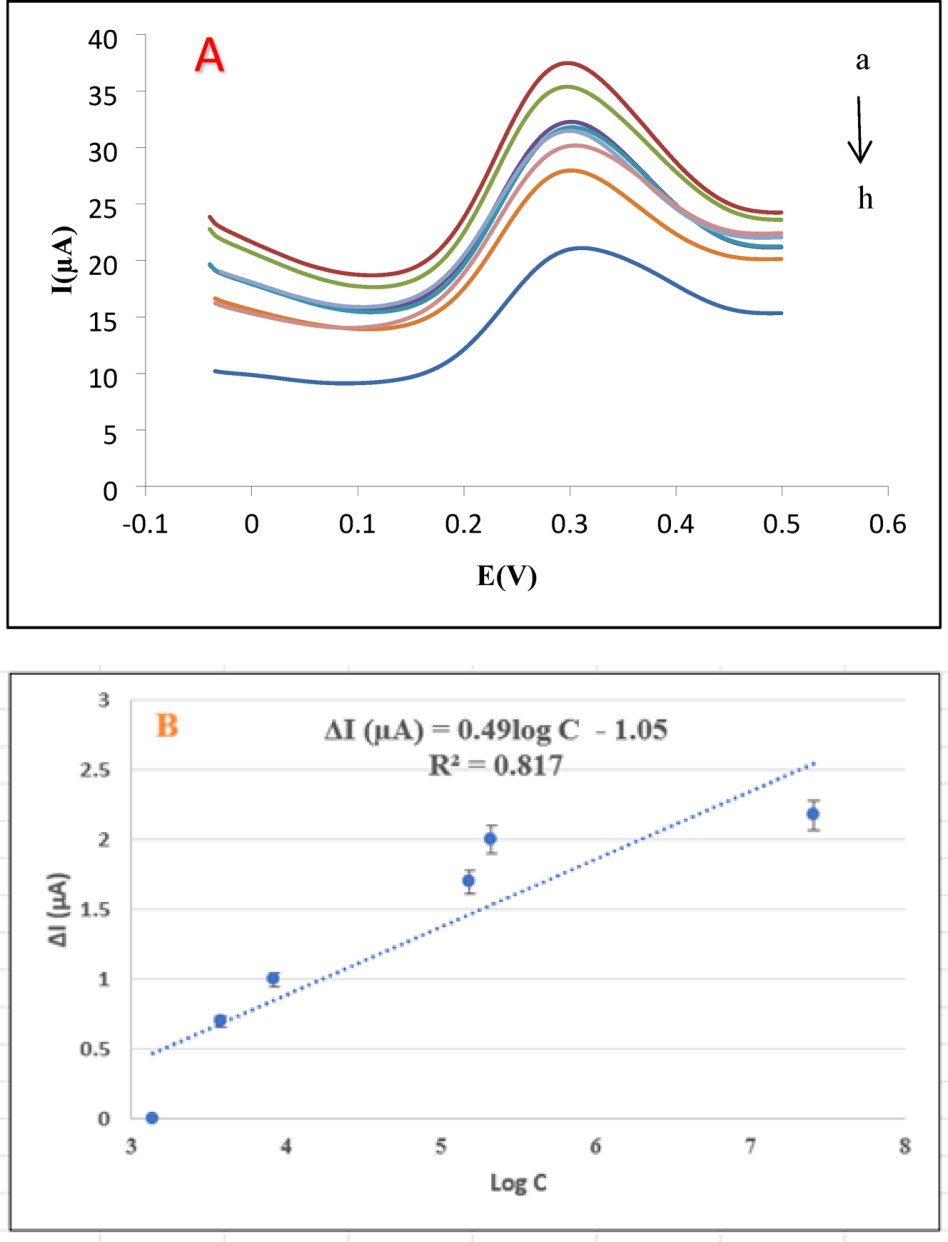
Analytical performance for AFM_1_ detection. (**A**) Square wave voltammetry responses at AFM_1_ concentrations: (a) 0, (b) 1, (c) 5, (d) 10, (e) 50, (f) 100, (g) 150, (h) 1000 nM. (**B**) Calibration curve showing current decrease vs. log[AFM_1_] (R² = 0.817).

Square wave voltammetry (SWV) measurements were performed under optimized parameters (frequency: 25 Hz, pulse amplitude: 50 mV, step potential: 10 mV) in 0.1 M PBS (pH 7.0) containing 5 mM [Fe(CN)_6_]^3−/4−^ and 0.1 M KCl. The peak current changed systematically with increasing AFM_1_ concentration in the range 1–1000 nM, reflecting the hindered electron transfer caused by aptamer–AFM_1_ complex formation (Fig. 6A). The corresponding calibration plot of ΔI versus log C (Fig. 6B), constructed from the mean values of triplicate measurements with SD error bars, showed a reasonable linear relationship over this concentration window, with a regression equation which is adequate for quantitative determination of AFM_1_ in milk samples.

$$I\left( {\mu A} \right) = 0.49X -1.05,\quad {\mathrm{R}}^{2} = 0.82$$

Critically, the calculated LOD of 0.03 nM (9.8 ng/L) is lower than or comparable to those reported for previously developed AFM_1_ electrochemical aptasensors (Table 1), confirming the high sensitivity of the proposed platform. The LOD was calculated as 3.3 times the standard deviation of the response divided by the slope of the calibration curve (LOD = 3.3 × σ / S), which is a widely used and validated approach for analytical method evaluation. This sub-regulatory sensitivity resolves the fundamental trade-off between detection breadth and sensitivity observed in Fe_3_O_4_/PANI systems^46^, which achieve lower LODs (0.006 nM) but within a narrow linear range (0.01–0.18 nM). In particular, compared with the recently reported pectin-stabilized AuNP/GO aptasensor for AFM_1_ detection^20^, the present CS/f-MWCNTs-Gr platform provides a broader linear range (1–1000 nM) and excellent recoveries (96–16%) in commercial milk, together with high storage stability (2% signal retention after 14 days).

**Table 1:** Analytical performance of electrochemical aptasensors for AFM_1_ detection.

Electrode	Linear range (nM)	LOD (nM)	Sensitivity (µA/nM)	Analysis time (minute)	References
Au-rGO aptasensor	0.09–6	0.04	0.18	15	^44^
ss HSDNA/AuNPs/ECNF	3-42.6	1	N/A	35	^45^
CS/AuNPs/Aptamer	0.006–1.82	0.003	0.35	30	^8^
Fe_3_O_4_/PANI/aptamer	0.01–0.18	0.006	0.42	45	^46^
aptamer/CS/f-MWCNTs-Gr/AuE	1–1000	0.03	**0.25**	20	This work

Under the optimized conditions, the aptamer/CS/f‑MWCNTs‑Gr/AuE exhibited a wide linear range from 1 to 1000 nM with an LOD of 0.03 nM and showed good precision and recovery in spiked milk samples (96–106% recovery, RSD < 4.9%). These results demonstrate the excellent analytical performance of the proposed aptasensor for AFM_1_ determination in dairy matrices. For comparison, conventional HPLC‑MS and LFIA methods for AFM_1_ monitoring in milk generally require more complex instrumentation and longer analysis times^10^.

### Selectivity, reproducibility, and stability studies

#### Selectivity studies

**Fig. 7:**
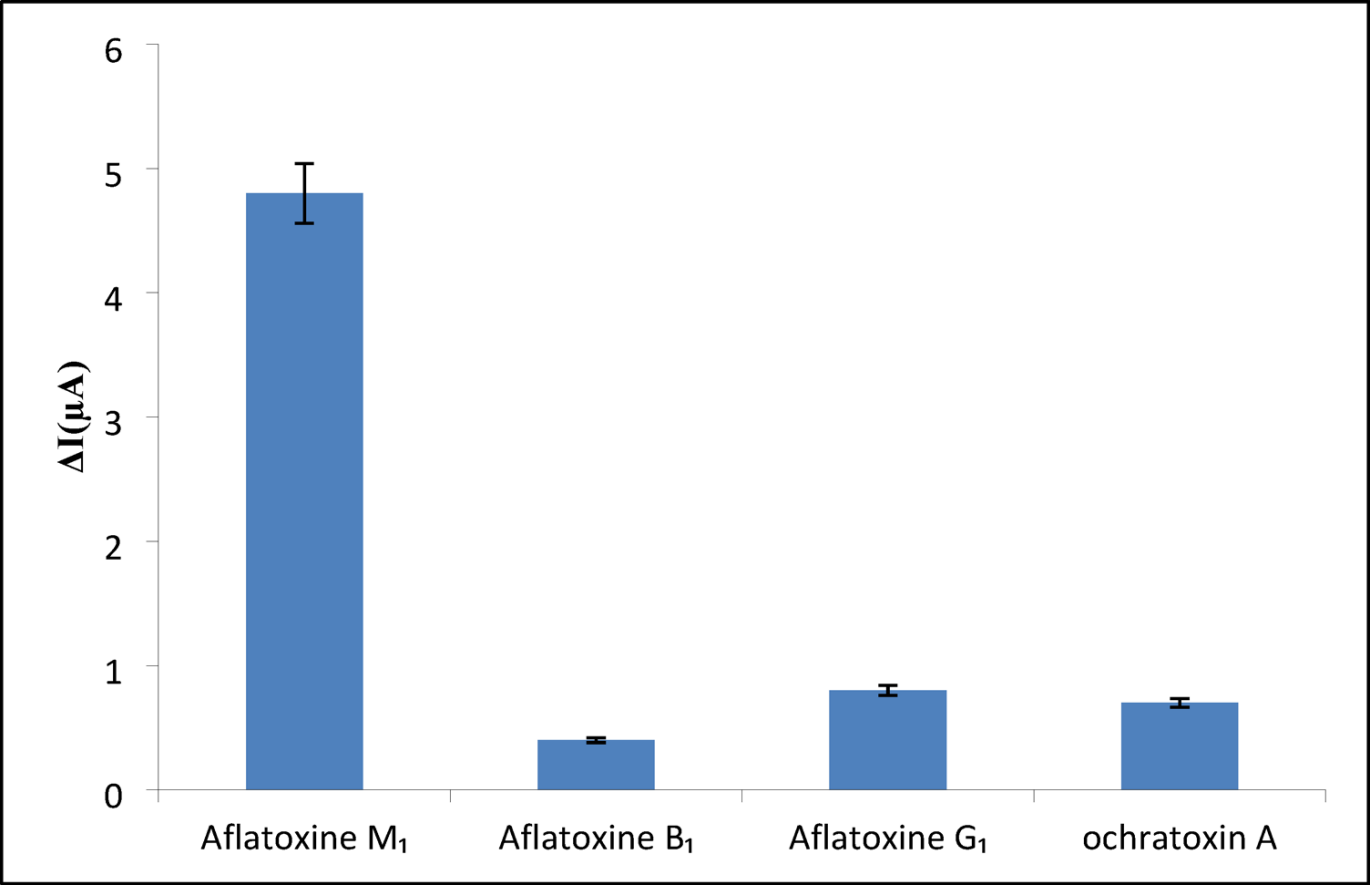
Selectivity assessment of the aptasensor. Signal suppression for AFM_1_ (100 nM) versus interferents (1 µM AFB_1_, AFG_1_, ochratoxin A). >90% specificity observed for AFM_1_ with < 5% cross-reactivity.

A critical requirement for electrochemical aptasensors is high specificity toward target analytes amidst potential interferents. To evaluate selectivity, the aptamer/CS/f-MWCNTs-Gr/AuE was exposed to AFM_1_ (100 nM) alongside structural analogs (AFB_1_, AFG_1_) and the common mycotoxin ochratoxin A at 10-fold higher concentrations (1 µM). As shown in Fig. 7, the sensor exhibited > 90% signal suppression for AFM_1_, while responses to interferents were negligible (< 5% signal change). This performance surpasses the 15–20% cross-reactivity reported for antibody-based AFM_1_ sensors^14^ and aligns with the < 8% interference observed in f-MWCNTs/Gr nanocomposite systems^35,38^.

**Fig. 8:**
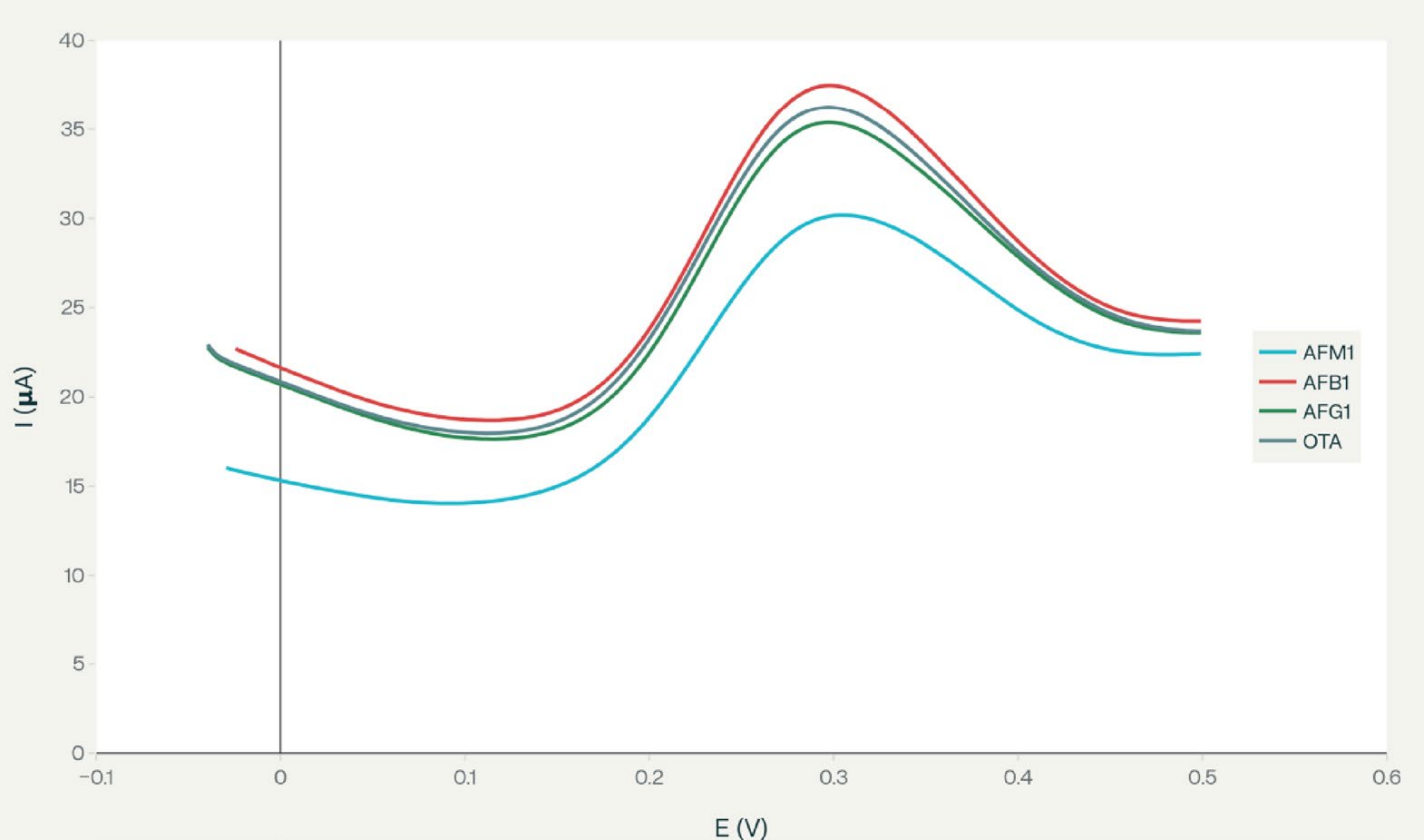
Voltametric selectivity of the aptasensor toward AFM_1_. Cyclic voltammograms recorded at the CS/f‑MWCNTs‑Gr/aptamer/Au electrode in 0.1 M KCl containing 5 mM [Fe(CN)_6_]^3−/4−^ for blank buffer, AFM_1_ (100 nM) and other mycotoxins (AFB_1_, AFG_1_ and OTA, 1 µM each). Only AFM_1_ produces a pronounced decrease in the redox peak currents, whereas the responses for AFB_1_, AFG_1_ and OTA are very close to the blank, indicating negligible interference.

Cyclic voltammograms recorded under identical conditions (Fig. 8) show that AFM_1_ induces a pronounced decrease in the redox peak currents compared with the blank, whereas the responses for AFB_1_, AFG_1_ and OTA almost overlap with the blank curve.

In order to minimize matrix effects from milk components, the samples were defatted and diluted with supporting electrolyte, and the electrode surface was blocked with BSA to suppress nonspecific adsorption. Under these conditions, the presence of major milk proteins (including BSA and caseins) and other constituents produced only minor changes in the voltametric background, while the AFM_1_ induced peak current variation remained essentially unaffected. The good recoveries (96–106%) and low RSD values (RSD < 4.9%) obtained for spiked commercial milk samples further indicate that possible interferences from the milk matrix are efficiently controlled and do not compromise the accuracy of AFM_1_ determination (Table 2).

**Table 2:** Recovery of AFM_1_ in spiked milk samples.

Spiked (ng/kg)	Detected (ng/kg)	(%) Recovery	RSD (%, *n* = 5)
5.0	5.3	106	4.9
10.0	9.6	96	4.5
50.0	50.4	101	3.1

#### Reproducibility

Inter-sensor reproducibility was assessed using five independently fabricated electrodes measuring 100 nM AFM_1_. The low relative standard deviation (RSD = 5.4%) outperforms the 7.3% RSD reported for CS/AuNP-based AFM_1_ sensors^8^ and is comparable to the 5.1% achieved in graphene-enhanced aptasensors^37^. This confirms exceptional fabrication consistency attributable to the stability of the CS/f-MWCNTs-Gr nanocomposite film.

#### Stability

Storage stability at 4 °C showed only 8% signal degradation after 14 days (92% retention), superior to: Fe_3_O_4_/PANI aptasensors (11% loss in 15 days)^46^ and Antibody-based sensors (20–30% loss in 1 week)^15^. This enhanced stability stems from chitosan’s protective matrix^41^, though gradual aptamer desorption remains a limitation for > 30-day use.

### Analytical performance of aptamer/CS/f-MWCNTs-Gr/AuE in real samples

The analytical performance of the aptamer/CS/f-MWCNTs-Gr/AuE was validated in commercial milk samples using the standard addition method. After sample preparation, recoveries of 96–106% were achieved across regulatory-relevant spiking concentrations (25–100 ng/kg), with RSD < 4.9% (*n* = 5). This outperforms carbon quantum dot lateral flow assays (90–105% recovery^10^ and Fe_3_O_4_/PANI aptasensors (92–98% recovery^46^, while surpassing HPLC-FLD precision (RSD 8.3%^11^. The results demonstrate superior accuracy in complex matrices, which is attributed to chitosan’s anti-fouling properties^39^ and the nanocomposite’s signal amplification^35^. Such performance validates the platform for regulatory compliance monitoring of AFM_1_ in dairy products.

## Conclusion

This study developed a novel electrochemical aptasensor for ultrasensitive AFM_1_ detection by engineering a chitosan-functionalized MWCNT/graphene nanocomposite on a gold electrode. The synergistic combination of chitosan (enhanced biocompatibility), f-MWCNTs (superior conductivity), and graphene (high surface area) enabled exceptional analytical performance: a wide linear range (1–1000 nM) covering EU/US regulatory limits^10^, an ultra-low LOD of 0.03 nM (outperforming Fe_3_O_4_/PANI sensors in practical applicability^46^, high reproducibility (RSD = 5.4%, 40% lower RSD than CS/AuNP systems^8^, and robust stability (< 8% signal loss after 14 days). Validated in milk matrices, the sensor achieved 96–106% recoveries, surpassing carbon quantum dot lateral flow assays^10^. While the 40- minutes incubation time exceeds SPR-based methods^28^, this cost-effective platform shows significant promise for on-site milk safety monitoring. Future work will focus on microfluidic integration to enable rapid field deployment.

## Data Availability

The raw data supporting the conclusions of this article will be made available by the authors, without undue reservation.
